# Development and clinical deployment of an automated planning tool for prostate only and male whole pelvis plans based on multi‐criteria optimization

**DOI:** 10.1002/acm2.70598

**Published:** 2026-05-04

**Authors:** Kai Huang, Kai Wang, Adam Schrum, Eric Kusmaul, Erica Fisler, Mariana Guerrero

**Affiliations:** ^1^ Department of Radiation Oncology University of Maryland Medical Center Baltimore Maryland USA; ^2^ Fred Hutchinson Cancer Center University of Washington School of Medicine Seattle Washington USA; ^3^ Department of Radiation Oncology University of Colorado School of Medicine Aurora, CO USA; ^4^ Department Radiation Oncology Central Maryland Radiation Oncology Center Columbia Maryland USA; ^5^ Department Radiation Oncology Baltimore Washington Medical Center Radiation Oncology Glen Burnie Maryland USA; ^6^ Department Radiation Oncology Kaufman Cancer Center Upper Chesapeake Medical Center Bel Air Maryland USA; ^7^ Department of Radiation Oncology University of Maryland School of Medicine Baltimore Maryland USA

**Keywords:** auto‐planning, multi‐criteria optimization, prostate cancer, radiotherapy, treatment planning

## Abstract

**Background:**

Multi‐criteria optimization (MCO) is an advanced optimization technique that can be applied to any problem with multiple objectives that may be conflicting. MCO has been available in commercial treatment planning systems (TPS) for several years now and has been applied to treatment planning of many anatomical locations in a variety of ways. The MCO optimization method is based on the Pareto plans generation and is very powerful, but there are significant hurdles in terms of clinical implementation due to long computing times, lack of dosimetrists training and plan degradation after the optimized fluence is converted to deliverable. While some authors have studied the use of MCO in automation, no clinical implementation of an MCO‐based auto‐planning technique has been reported.

**Purpose:**

This study aims to develop and clinically deploy an automated planning tool based on MCO for prostate and whole‐pelvis radiotherapy.

**Materials and methods:**

A Python script based on a commercial treatment planning system was developed to automate MCO, including Pareto plan generation, fluence plan selection, dose conversion, and post‐processing. The tool underwent retrospective validation on 10 prostate patients with the input of four dosimetrists and a 10‐month prospective pilot involving another three senior dosimetrists across different community sites. Dosimetrists evaluated plan quality and provided quantitative and qualitative feedback for iterative improvements of the tool. The study reports on the plan comparisons between the clinical and the MCO generated plans for retrospective patients. The study also reports the prospective use cases and the qualitative and quantitative evaluations from dosimetrists.

**Results:**

Retrospective evaluations showed 82.5% of MCO prostate plans were clinically acceptable. The tool generated prostate plans in approximately 10.1 min and whole pelvis plans in 27.2 min. Dosimetric analysis revealed comparable plan quality to clinical plans, with MCO plans achieving lower organ‐at‐risk doses. In the pilot phase, the MCO tool was used for 41 prospective patients, producing plans that dosimetrists could refine to achieve clinical acceptability within a median of 10 min.

**Conclusions:**

This study demonstrates the successful development and clinical implementation of an MCO‐based automated planning tool for generating acceptable VMAT plans for prostate and whole pelvis radiotherapy. The extensive pilot phase showcases an effective strategy for integrating automated planning solutions into routine clinical practice.

## 1 INTRODUCTION

Radiotherapy treatment planning requires balancing competing objectives—delivering sufficient dose to the tumor while minimizing exposure to surrounding organs at risk (OARs). This trade‐off has traditionally been managed through an iterative, manual process performed by experienced dosimetrists and physicists. However, manual planning is time‐consuming, and plan quality can vary significantly depending on the planner's expertise and the complexity of the case.^1^, ^2^ As the demand for high‐quality, personalized radiotherapy continues to grow, the development of tools capable of efficiently generating clinically acceptable, high‐quality plans has become increasingly important.

Multi‐criteria optimization (MCO) provides a framework to approach this problem, generating a set of Pareto‐optimal solutions that represent different trade‐offs between objectives.^3^, ^4^ Among commercial treatment planning systems (TPS), RayStation (RaySearch Laboratories, Stockholm, Sweden) was the first to offer an MCO module for clinical use, enabling planners to interactively navigate these solutions. Despite its promise, the broader clinical adoption of MCO has been limited due to the labor‐intensive nature of generating, navigating, and converting Pareto plans into deliverable dose distributions.^4^, ^5^, ^6^, ^7^

Simultaneously, the field of auto‐planning has made significant strides, particularly with the advent of knowledge‐based planning (KBP) and deep learning‐based methods.^1^, ^8^, ^9^, ^10^, ^11^, ^12^ These approaches aim to standardize and expedite plan generation, often demonstrating reductions in planning time and inter‐planner variability. However, these knowledge and model‐based approaches face several challenges: they rely heavily on large, curated datasets; performance may degrade when applied to anatomy not represented in the training set; and retraining is often required when clinical practice guidelines evolve.^11^, ^12^, ^13^, ^14^ Moreover, many AI‐based auto‐planning systems function as “black boxes,” limiting interpretability and user control in clinic.^15^, ^16^

An alternative strategy to machine learning‐based auto‐planning is to automate the MCO process itself—leveraging Pareto solutions generated for the individual patient rather than relying on historical data. This has the advantage of being model‐free, inherently interpretable, and fully customizable based on clinical goals. While MCO navigation has typically been a manual step, recent work has shown that automatic selection of a balanced Pareto plan, in conjunction with post‐processing algorithms, can yield clinically acceptable plans.^17^, ^18^ However, there is limited literature on the full clinical deployment of such an automated MCO‐based workflow, particularly in multi‐clinic‐site settings with variable user expertise and physician preferences.

In this work, we describe the development and clinical deployment of a fully automated treatment planning tool for volumetric modulated arc therapy (VMAT) using MCO in RayStation. The tool was designed to generate prostate‐only and male whole‐pelvis plans using a standardized, institution‐wide set of dosimetric objectives and constraints. The automated workflow includes Pareto plan generation, selection of a balanced fluence solution, dose conversion, and multi‐pass post‐processing, implemented through a Python‐based scripting interface. Importantly, the approach requires no prior training data and is not based on previously treated patients, distinguishing it from knowledge‐based or AI‐driven approaches.

Prostate cancer was selected as the initial application site due to its high incidence, relatively consistent target and organ‐at‐risk anatomy, and the substantial planning workload it represents in daily practice.^19^, ^20^ These characteristics make it one of the ideal starting points for evaluating and deploying automated planning solutions with potential for broad clinical impact.

## 2 MATERIALS AND METHODS

Our project can be divided into two main parts. The first part involves developing and validating a fully automated MCO planning process using RayStation script capabilities. In this phase, we tested 10 retrospective single prostate plans and gathered feedback from the dosimetry team to evaluate and improve the script to meet our institution's planning guidelines. In addition, to enable a dosimetric comparison between clinical plans and the automated plans, we applied the final version of the script to 19 retrospective single prostate patients and collected the resulting data. The clinical plans are optimized in Raystation using the Direct Parameter Optimization algorithm (DMPO) without using the manual MCO workflow.

The second part is the clinical release, which was deployed in two phases: a pilot phase involving the limited clinical release of the automated script to a group of three experienced dosimetrists. During this phase, the script was further refined and debugged, and the first 20 patients were treated with automatically generated plans after review and improvements made by dosimetrists if applicable and approval by physicians. The second phase is the widespread clinical release to the whole department which is currently being monitored and remains ongoing. The automation for whole pelvis plans was added to the script capability and underwent the same testing and evaluation process. Below we present the details of the two parts of our projects.

### 2.1 MCO process automation, validation and dosimetric evaluation

#### 2.1.1 MCO process automation

The process of MCO manual planning can be divided into 3 main stages: Pareto plan generation, navigation, and post‐processing. First, the Pareto plans were generated based on a set of objectives and constraints given by users. In RayStation, the Pareto plans use a pencil beam algorithm for dose calculation and fluence‐based optimization. The more Pareto plans are generated, the more time and computational resources it takes. Second, the user “navigates” by changing various weightings of the generated Pareto plans to select the desired dose distribution. This step is often performed manually by dosimetrists or physicians. Our automated process omits the manual navigation by choosing the balanced fluence plan as explained below. Lastly, once the navigated plan is obtained, the dose must be converted into the final dose calculation, in our case the Collapsed Cone Convolution superposition algorithm from RayStation. During the conversion, the plan quality often degrades, and to bring it back to an acceptable level, a subsequent optimization is needed. This additional and final tweak of the plan is called post‐processing, and it is performed using the standard Direct Machine Parameter Optimization algorithm from RayStation using the MCO plan as a “mimic dose” objective.

**FIGURE 1:**
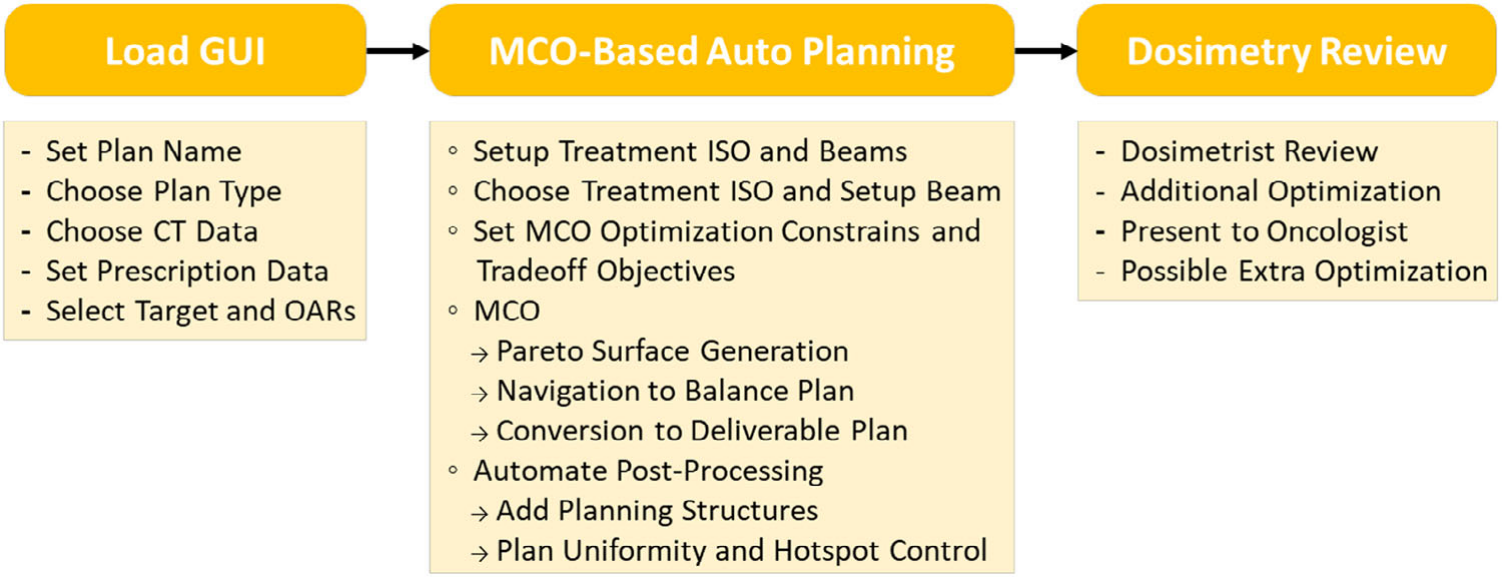
Flow chart of the process for using the proposed MCO‐based automated planning tool. Steps start with “‐” indicates manual procedure, “°” and “→” indicates automatic procedure.

To automate the MCO planning process, each of these steps must be automated and executed in sequence using a Python‐based script utilizing RayStation application programming interface (API) library. Figure 1 shows the process of using the MCO tool with different symbols corresponding to the steps that are automated versus the steps that are done manually by dosimetrists.

To automatically generate Pareto plans, our study used one set of objectives and constraints for each anatomical site, prostate only and whole pelvis. The sets of objectives and constraints were built upon a previous report from the literature^17^ and were chosen and evaluated based on the planning goals established by our institution's clinical practice guidelines that are regularly updated every three years. Table 1 lists the specific objectives and constraints used for both prostate and whole pelvis MCO plans. All plans had two full arcs, with collimator angles set to 10° and 350°, and no couch kicks. The isocenter was consistently placed at the center of the planning target volume (PTV). The use of EUD = 0 with parameter *A* = 1 for these critical structures is based on the recommendation of the vendor for MCO planning.

**TABLE 1:** A universal set of constraints and objectives used for creating the Pareto plans. Prostate and whole pelvis (WP) indicate the constraints and objectives are specific for prostate only or whole pelvis, respectively.

Constraints	
**PTV**	Min dose 95%Rx
**PTV**	Max dose 105%Rx
**Prostate**	Min dose 100%Rx
**Seminal Vesicle (Prostate)**	Min dose 100%Rx
**CTV (WP)**	Min dose 100%Rx

Trade‐off objectives	
**PTV**	Uniform dose 100%Rx
**Rectum**	Max EUD 0 cGy, Parameter A 1
**Bladder**	Max EUD 0 cGy, Parameter A 1
**External**	Dose fall‐off 95%Rx to 50%Rx, distance at 0.6 cm
**External**	Dose fall‐off 50%Rx to 0%, distance at 10 cm
**Small bowel (WP)**	Max EUD 0 cGy, Parameter A 1
**Large bowel (WP)**	Max EUD 0 cGy, Parameter A 1

Our study omitted the necessity of manual navigation by electing to use the balanced fluence plan generated by giving equal emphasis to all objectives. The balanced fluence plan was then converted into a deliverable dose distribution with leaf sequence. Targets and OARs were defined as user‐specified regions of interest (ROIs) to focus the dose mimicking optimization used by RayStation during the conversion on these regions. The step for dose mimicking optimization was continued 2 times for prostate plans and 5 times for whole pelvis plans to obtain the deliverable plan closest to the navigated plan.

**FIGURE 2:**
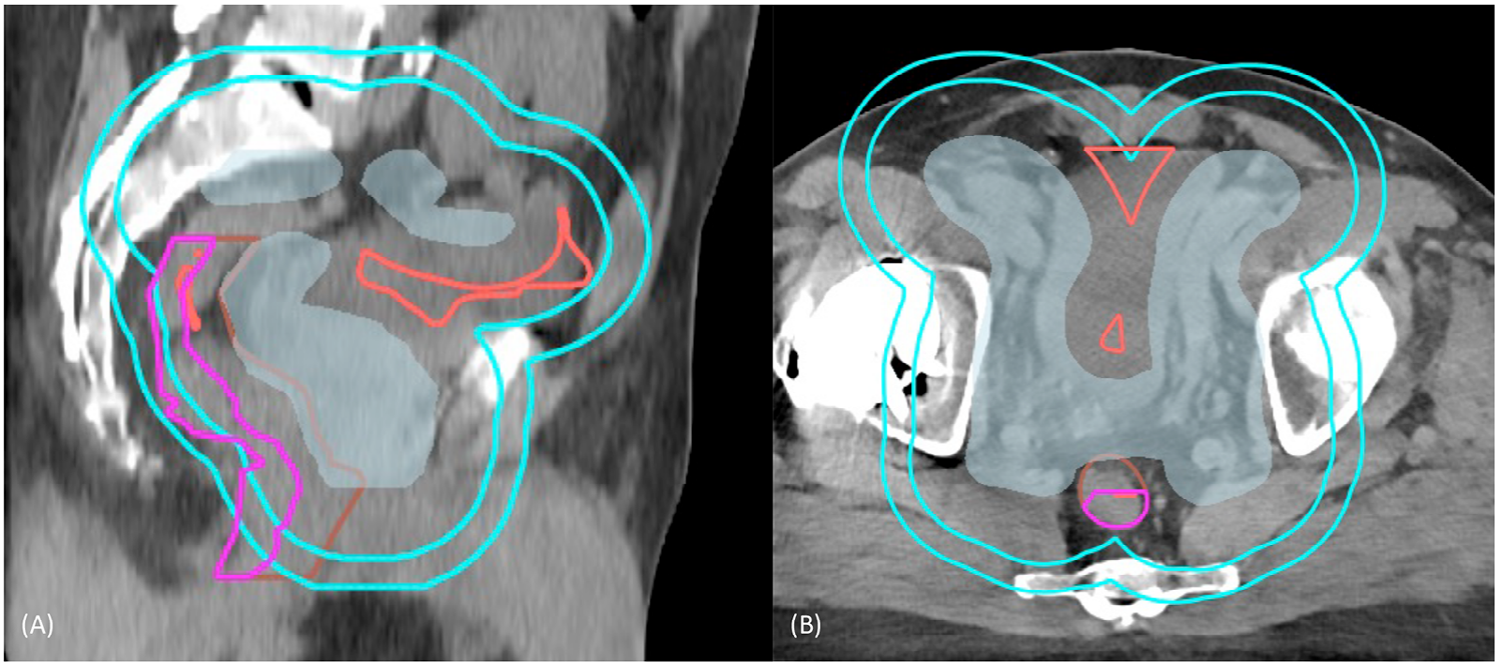
The automatically generated planning structures. Magenta is the planning structure for the posterior half of the rectum minus the PTV expanded uniformly by 7 mm. Cyan is the PTV ring structure. Red is the overlap of the PTV expanded laterally by 15 cm, excluding the PTV expanded uniformly by 1 cm. The overlap structure may extend to the posterior side if the PTV slices have a concave shape in the posterior direction. Shaded blue is the PTV. Brown is the rectum.

Subsequently, a list of post‐processing objectives was applied to compensate for the plan degradation during the conversion from fluence to deliverable dose and further refine and control the dose falloff in the final plan. To fine tune the deliverable plans and further reduce dose falloff outside of the targets, specific planning structures were created and employed (Figure 2). For prostate plans, a ring structure was created by subtracting PTV expanded uniformly by 2 cm from the PTV expanded uniformly by 3 cm. This ring structure is set to a max DVH objective targeting half of the prescription dose to 0 cc, intending to control the dose spillage around PTV. Additionally, a structure representing the posterior half of rectum minus the PTV expanded uniformly by 7 mm was created to specifically control the 50% prescription dose isodose line's coverage of rectal slices. For the whole pelvis plans, two additional objectives were incorporated. The first one is the PTV uniformity objective with its relative standard deviation of 1.5%. The second objective applies the max dose of 60% of prescription dose to a planning structure constructed as follows: first we expand PTV laterally to the right by 15 cm and calculate the intersection with the PTV expanded laterally to the left by 15 cm. Then we subtract the planning structure by PTV with 1 cm uniform expansion. The resulting structure helps control the excess of dose, that systematically appears after the conversion to a deliverable plan centrally, in‐between the pelvic nodes. All post‐processing objectives were scaled according to the dose reference function designed to mimic the MCO dose distribution. For both prostate and whole pelvis plans, the final dose optimization was continued for two iterations. The MLC leaf motion was constrained as 0.46 cm/deg. The additional structures created and optimization in the post‐processing steps are needed to address the plan quality degradation after the conversion to a deliverable plan from the fluence‐based plan. These steps cannot be avoided in the current version of RayStation dose calculation models, and the degree and location of dose degradation cannot be anticipated during the Pareto plan creation.

An in‐house, Python‐based tool was developed based on RayStation 11B platform to automate the VMAT plan creation process described above. A graphical user interface (See Figure S1) was devised to facilitate effective use by dosimetrists. The tool allows dosimetrists to specify the plan name, select the CT dataset, and identify the necessary target volumes and ROI names for structure creation.

#### 2.1.2 Validation by the dosimetry team

To validate the MCO automation tool, a group of four experienced dosimetrists from different community sites with physicians that have their own personal preferences was selected. For the prostate planning functionality, ten MCO plans were generated for retrospective, low‐risk prostate cancer patients and evaluated by the four dosimetrists using a 4‐point scale: 4 ‐ use‐as is, 3 ‐ acceptable needing physician‐preferred changes, 2 ‐ needs further optimization, 1 ‐ needs re‐plan. Plans scoring 3 or 4 were considered acceptable. The dosimetrists’ initial evaluations are shown in the results.

#### 2.1.3 Quantitative evaluation of plans dosimetry and efficiency

To provide quantitative evaluations, we report the DVH and dosimetric comparison between the final version of the automated tool's plans and the clinically approved plans for 19 retrospective prostate patients and 8 retrospective whole pelvis patients. The whole pelvis patients consistently received 4500 cGy in 25 fractions. The prostate‐only patients received various fractionation schemes ranging from 6000 cGy to 7920 cGy, with the most common being 6000 cGy in 20 fractions (*n* = 10) and 7000 cGy in 28 fractions (*n* = 7). The automated plans used the same fractionations as the clinical plans for the ease of comparison and for testing the robustness of the tool to changes in fractionations. For consistent comparison, all plans were normalized using the same normalization as the clinical plans. Our dosimetrists normalize plans to PTV such that D95% (dose to 95% of the volume) is 95% of the prescription dose at minimum and the CTV D95% to 100% of the prescription dose. Details of the dosimetric parameters can found in the supplementary materials. Statistical analysis was performed using the Wilcoxon signed‐rank test to determine if the dose metrics from the MCO‐generated plans were significantly different from the clinical plans.

### 2.2 Clinical testing and deployment

The project was approved by our institution's IRB. We are part of a large institution with several community sites and a large physician and dosimetry group. The tool underwent extensive validation and a pilot phase started in July 1^st^ 2024 before widespread clinical release on April 10th, 2025.

The pilot phase involved a limited clinical released with three senior dosimetrists from three different community sites. Senior dosimetrists with varied experience, exposure to different prostate patient populations and who have interests in the project were deliberately recruited to enhance the diversity of the pilot testing. The whole pelvis planning functionality was added to the tool subsequently and released for pilot use.

The goal of the pilot period is to generate enough data and confidence in the functionality of the MCO tool on at least 20 live patient cases per site before being released to clinic. Each generated plan was evaluated and iteratively improved by dosimetrists. Dosimetrists participating in the pilot were asked to complete a survey documenting the time spent on plan adjustments, their qualitative assessment of the plan quality, and their quantitative evaluation using the previously mentioned four‐point scale. All plans were evaluated in accordance with our institution's clinical practice guidelines. Over the 10 months of piloting, the tool was further refined, addressing bugs and improving the post‐processing algorithms based on dosimetrist feedback. The finalized version of the tool was then released for clinical use in April 2025.

The results will report data from the pilot study, specifically the automated planning time and the additional time reported by dosimetrists for further plan improvements needed before presenting the plans to attending physicians.

## 3 RESULTS

### 3.1 Validation by dosimetry team

The validation evaluation of the 10 retrospective prostate patients by four independent dosimetrists is presented in Table 2. On average across these evaluations, 82.5% of the MCO‐generated plans were deemed acceptable (scoring 3 or 4). For plans scored as 2, the comments were that rectal dose were too high, more than 105% of the prescription dose and prescription dose covering outside of PTV. For the plans scored as 3, dosimetrists would like to see 50% isodose lines not encompassing the entire rectum at each slice and hotspot being near OARs such as rectum and bladder. Overall, the dosimetrists participating in reviewing the plans gave favorable comments to MCO‐generated plans and would be willing to incorporate the MCO‐generated plans into their clinical workflow. This favorable result allowed us to further fine tune the algorithm based on the feedback, initiate the pilot phase and begin recruitment of senior dosimetrists for the testing of prostate and whole pelvis functionality.

**TABLE 2:** Dosimetrists review of 10 single prostate plans retrospectively before the tool was used for pilot phase.

	Score				Summary	
Dosimetrist	4	3	2	1	Acceptable	Minor edits
**1**	4	4	2	0	80%	20%
**2**	3	6	1	0	90%	10%
**3**	0	7	3	0	70%	30%
**4**	3	6	1	0	90%	10%
				Avg.	82.5%	17.5%

### 3.2 Quantitative evaluation of dosimetry and efficiency in retrospective patient cohort

The following results apply to the final version of the MCO generated plans versus the clinical plans on the cohort of the 19 retrospective prostate plans and 8 whole pelvis plans. Regarding planning efficiency, the final version of the automated tool generated prostate plans in an average of 10.1 ± 1.1 min and whole pelvis plans in an average of 27.2 ± 3.1 min. In terms of monitor units (MU), whole pelvis MCO plans required 11% more MU on average compared to clinical plans, while prostate MCO plans required 3.2% less MU. Analysis of hotspots indicated that MCO prostate plans were, on average, 1.85% hotter than clinical plans based on the dose to 0.03 cc within an external ROI. For whole pelvis plans, MCO plans were 1.1% hotter on average than the clinical plans.

**FIGURE 3:**
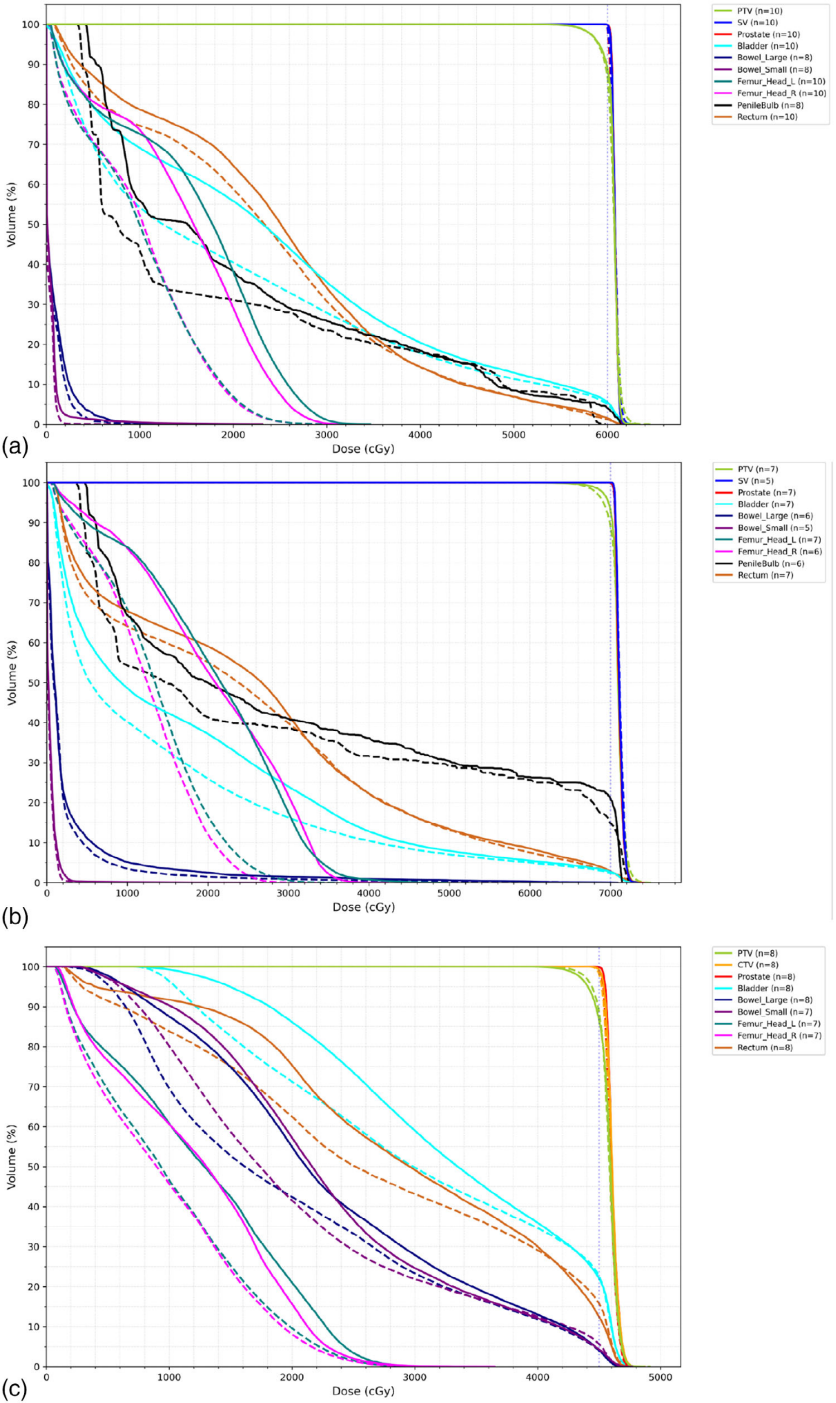
The mean DVH comparison between clinical (solid) and MCO (dashed) plans for prostate plans with prescription of (a) 6000 cGy and (b) 7000 cGy, and (c) whole pelvis plans with prescripton of 4500 cGy. SV stands for seminal vesicle. The number of DVH used to calculate the mean DVH is shown in the paranthesis of the legend indicated by *n*.

Dosimetric comparisons between the initial retrospective MCO and clinical plans are detailed in Supplementary material (Table S1 and Table S2 present dose metrics for prostate only plans and Table S3 for whole pelvis plans). Figure 3 shows the mean DVH comparison between clinical and MCO plans for prostate plans, both 6000 cGy and 7000 cGy total prescription dose, and whole pelvis plan for 4500 cGy in 25 fractions. The results from other fractionation schemes (each with *n* = 1) were consistent with the presented findings thus the dose metrics comparison are not presented due to small sample size. Most dose metrics were not statistically significantly different between the MCO and clinical plans. However, for certain metrics where a significant difference was observed, MCO plans consistently showed markedly lower doses to surrounding OARs including the femoral heads, penile bulbs, and lower average and minimum doses to the bladder. Overall, the MCO plans are dosimetrically comparable with the clinical plans in terms of plan quality.

### 3.3 Pilot phase time and plan qualitative evaluation by dosimetry on prospective patients

**FIGURE 4:**
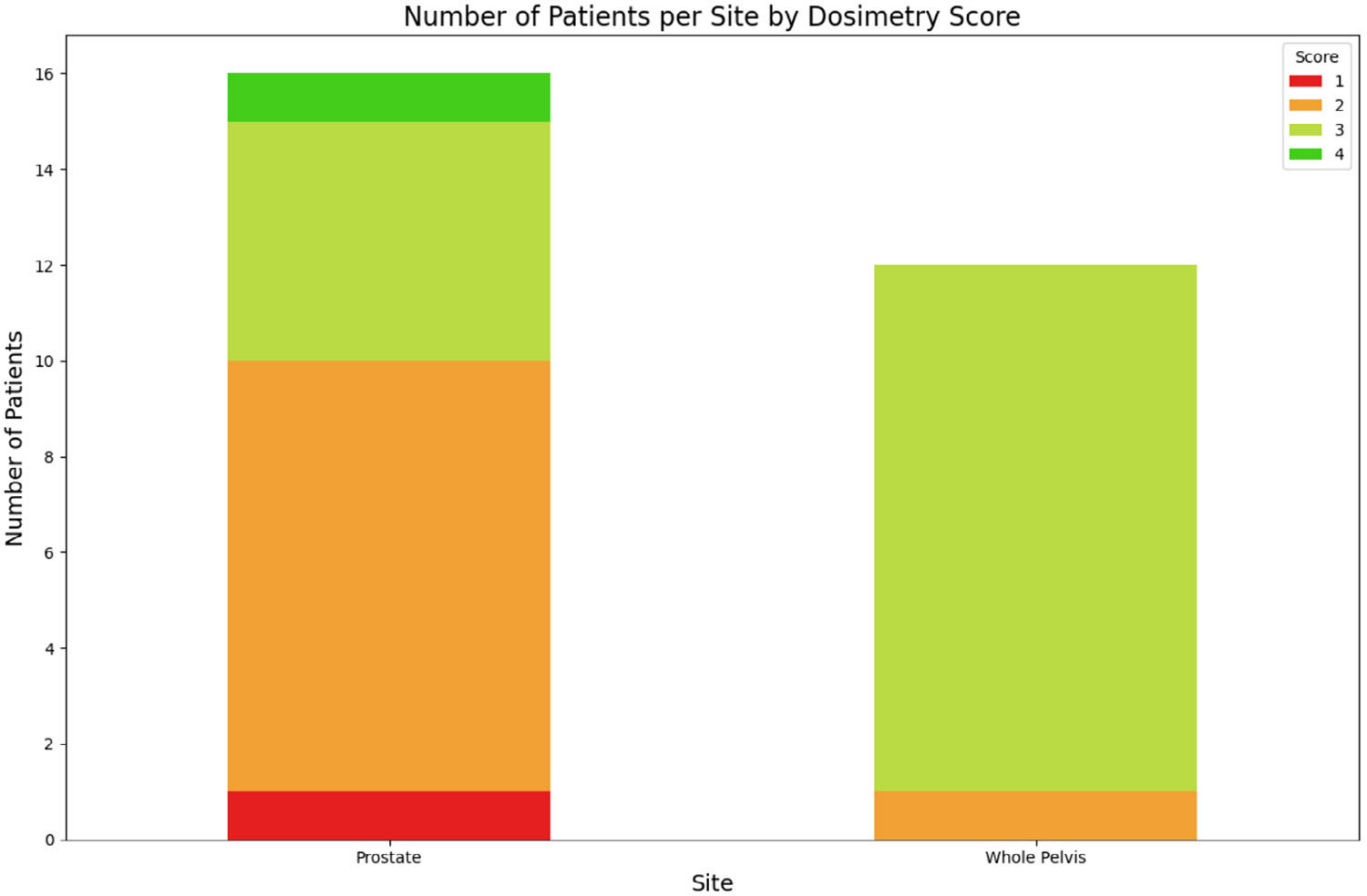
Number of patients per site by dosimetry scoring based on the 4‐point scale.

Data from our pilot study on live patients, conducted across three community sites, showed that the prostate‐only tool was used for 20 live patients, generating 25 plans, while the whole pelvis tool was used for 21 live patients, generating 22 plans. Dosimetrists spent an additional 10 min on median (25%–75%: 10–22.5 mins) improving prostate plans and 10 min on median (25%–75%: 5–10 mins) improving whole pelvis plans after initial generation by the tool. The total time spent creating the plan with the script and adjusting the plans remained less than 30 min for both sites. Figure 4 shows the distribution of dosimetrist evaluation scores from the pilot phase, indicating that the majority of plans were scored as acceptable (3 or above). The single prostate plan requiring a re‐plan was for a patient with hip prosthesis, a now known contraindication for the tool which uses two full arcs instead of partial arcs.

Dosimetrists provided qualitative comments on the MCO‐generated plans. The most frequently mentioned observation was the presence of 105% of prescription dose hotspots located within OARs such as the bladder, rectum, or penile bulb, requiring adjustment to shift these hotspots towards the target. Plans scored as 2 receive the same general comments as the plans scored as 3, meaning hotspots are in OARs and need to be relocated. Therefore, the distribution of the scores between 2 and 3 is reflective of dosimetrists’ interpretation of what plans are acceptable with physician preferred changes (scored as 3) versus needing further optimization (scored as 2), based on their experience working with physicians.

Dosimetrists were able to successfully modify these plans using additional objectives to meet clinical standards for review by attending physicians. None of the plans required further replanning after attending physician reviews, and all passed calculation‐based patient‐specific quality assurance. Ultimately, these plans were approved and used for patient treatment. Beyond the noted hotspots, dosimetrists generally provided positive feedback on the quality of plans produced by the MCO tool, indicating that most required only minor modifications.

## 4 DISCUSSION

This study demonstrates the successful development and clinical deployment of an automated planning tool based on MCO for prostate‐only and male whole‐pelvis radiotherapy. The tool, implemented within the RayStation treatment planning system, automates each component of the MCO workflow—from Pareto plan generation to post‐processing—without relying on prior patient data or model training. Our findings support the clinical adoption of this model‐free, patient‐specific automation, offering an alternative to data‐driven approaches such as knowledge‐based or deep learning‐based planning that often require extensive and well‐curated training data.

Our findings showed that the automated MCO approach is non‐inferior to manual planning, yielding comparable dosimetric plan quality, while exhibiting superiority over clinical plans in most OAR metrics. We acknowledge the relatively small sample size is a limitation of our study, which may limit the generalizability of the statistical findings. However, the improvements in OAR doses demonstrate the tool's capacity for consistent optimization, effectively reaching the Pareto front to minimize OAR exposure while maintaining robust target coverage.

Importantly, the tool produced clinically acceptable plans for most retrospective and prospective patients, requiring only minor manual adjustments by dosimetrists. This consistency was maintained across multiple community sites with varied physician preferences and patient population, indicating robust generalizability. The tool reduced overall planning time, with median dosimetrist involvement under 10 min per case. The reduction in active manual planning is significant compared to manual planning time reported in literature ranging from 30 mins to 2.5 h.^6^, ^21^ Our work presents an automated treatment planning solution that is suitable for the majority of patients with little or no further modifications based on experienced dosimetrists evaluations. For patients where additional fine tunings of the dose were needed, typical objectives were removal of small remaining hot spots in PTV or OARs.

Our findings align with and extend prior work in MCO‐based treatment planning. Over the past decade, multiple strategies have been proposed to incorporate MCO into clinical radiotherapy planning. In an early foundational study, Craft et al. demonstrated that Pareto surface‐based MCO significantly reduced planning time and improved dosimetric quality for glioblastoma and locally advanced pancreatic cancer. Their implementation highlighted the potential of real‐time trade‐off navigation by clinicians to surpass conventional trial‐and‐error workflows.^5^, ^6^ McGarry et al. evaluated navigation‐based MCO for step‐and‐shoot IMRT in localized prostate cancer. While MCO achieved comparable target coverage and rectal sparing relative to standard plans, they noted that the transition from fluence‐based to deliverable plans introduced dose degradation, particularly in scenarios prioritizing organ‐at‐risk (OAR) sparing.^20^ Other authors extended the application of MCO to VMAT across multiple anatomical sites using site‐specific class solutions.^17^ Their study demonstrated the feasibility of using a universal objective set for each site, yielding plans dosimetrically comparable to those produced clinically, though it still required physician‐led navigation of the Pareto surface.^17^ More recently, Biston et al. assessed a fully automated a priori MCO solution (mCycle) for head‐and‐neck VMAT planning. Their results indicated superior plan quality over manual VMAT and Helical Tomotherapy planning; however, the implementation required separate wish lists for complex anatomical subgroups and was limited to a research version of their treatment planning system.^18^ In contrast, the current study demonstrates a fully automated, clinically available MCO‐based VMAT planning pipeline. By auto‐selecting a balanced Pareto plan followed by multi‐pass post‐processing, our method achieves high‐quality, clinically acceptable plans using a universal set of planning objectives and constraints.

There have been efforts to include information from Pareto solutions into auto‐planning techniques. Wheeler et al^22^, ^23^, ^24^ demonstrated that using a protocol‐based automatic iterative optimizations (PB‐AIO) it is possible to develop a Pareto‐guided methodology for auto‐planning using RayStation scripting for prostate patients. To achieve this, they developed an in‐house Pareto tool to dynamically changed the objectives for trade‐off goals in the optimization process to “minimize OAR doses and keeping trade‐off balancing across patient consistent”. The initial weights are “calibrated” in a learning set of patients, where manual navigation of the Pareto surface determines the ideal plan for each patient in the calibration set. In contrast, we use the previously developed set of universal MCO objectives and constraints^17^ for each anatomical site and relay on the commercially available RayStation Pareto plan generation tool. We always navigate to the balance plan and then use automated postprocessing after the conversion to deliverable plans with goals determined by trial and error in an initial small set of patients. Further individualization of the plan if it is needed can be done by the user, who can manually tweak any needed dosimetric parameters. Our approach does not require any deep learning capabilities as the automation is driven by the patient's specific Pareto plans and the use of the balanced plan for automatic navigation.

While MCO optimization is well‐documented in a research context, its routine application in a live clinical environment remains rare in the US. Our work demonstrated that MCO‐automated tools could be safe and seamlessly integrated to enhance planning efficiency without disrupting established clinical workflows. A key strength of this study is the 10‐month prospective pilot phase involving three senior dosimetrists from different community sites. The incorporation of dosimetrist quantitative and qualitative scoring to the evaluation process is critical as it bridges the gap between purely DVH evaluation and nuanced clinical judgements that dosimetrists bring. This pilot phase provided critical feedback for iterative tool refinement and ensured integration with existing clinical workflows. The structured pilot also served to build confidence in the tool's reliability, facilitating a smooth transition to clinical deployment. Unlike many auto‐planning studies focused solely on dosimetric endpoints, we emphasize the importance of clinical usability, institutional acceptance, and workflow preservation—factors essential for adoption and sustainable implementation in practice. The 10‐month recruitment period reflects a controlled pilot scope limited to three senior dosimetrists to ensure patient safety, as well as a concurrent increase in the clinical use of simultaneous integrated boost fractionation schemes that fell outside the tool's current scope, reducing the number of plans eligible for the study.

The tool's success relies on two main components: a standardized set of planning objectives and constraints, defined by institutional guidelines, and automation of the MCO process without manual navigation. An essential aspect of clinical integration is that all automated plans undergo the same evaluation, assessment, and modification process by dosimetrists before physician review, ensuring adherence to clinical standards. The inherent consistency and speed of generating MCO auto plans are particularly valuable for dosimetrists working under tight deadlines.

The automation of MCO without manual navigation departs from the conventional MCO methodology, which relies on user‐guided navigation of Pareto surfaces. Instead, we selected a balanced fluence plan, giving equal weight to all objectives, and refined the dose converted from the fluence plans using multiple post‐processing iterations. While the treatment planning system offers auto‐navigation tool, our preliminary testing was unsuccessful in using the provided tool to identify more idealized fluence maps. After extensive testing, we found that the strategy of using the balanced fluence plan was sufficient to produce clinically acceptable plans while eliminating the need for user input during intermediate steps—a key innovation that addresses a long‐standing bottleneck using MCO‐based planning in our clinical workflow. A more sophisticated auto‐navigation tool, beyond using balanced plan, to further improve the selection of fluence maps could be explored in future work.

In terms of limitations, we can identify the following. First, limitation of the script concerns the development of the universal sets of constraints and objectives based on the use of two full arcs. This approach makes the tool contra‐indicated in situations where full arcs must be avoided, such as for patients with prosthetic implants or cases too complex to achieve adequate dose distribution with only two arcs. Further investigation is needed to determine if incorporating more arcs and using user‐determined collimator angles could improve plan quality for such challenging scenarios. Second, the dose conversion step from fluence to deliverable plan remains a challenge. Plan degradation during this step necessitates post‐processing, and while our strategy of multiple conversion iterations is effective to some extent, it remains computationally intensive. The degradation during the conversion to deliverable should also be addressed, potentially implementing a direct aperture type of optimization by the vendor, which currently can only be used for the sliding window technique limiting the VMAT optimization degrees of freedom. Moreover, the current tool is built specifically on the RayStation TPS API, limiting its direct transferability to MCO products from other vendors without significant modification. However, the core principle of using a universal set of constraints and objectives to drive MCO auto‐planning is a concept that should be applicable regardless of the specific vendor platform or research‐based MCO planning algorithm. Additionally, the current version of the tool does not explicitly control the location of maximum dose and hot spots. As a result, dosimetrists have to further fine‐tune the plans when the location of maximum dose is within or close to OARs.

Another limitation lies in the evaluation of clinical acceptability. Although plans were reviewed by experienced dosimetrists and confirmed by attending physicians, inter‐physician variability and site‐specific preferences can influence judgment. While we intentionally incorporated feedback from diverse sites, future work could formalize this process further with quantitative metrics linked to clinical outcomes.

The current tool is specific to Varian TrueBeam and C‐Series linear accelerators. Due to inherent differences in hardware and multi‐leaf collimator design, such as Elekta's longer MLC travel distance than Varian's design, direct application to other machines may require further adjustments to the field size determination. We have identified this as a study limitation and consider the expansion of the tool to other vendor's linear accelerator model an objective for future studies.

For future work, we plan to explore the application of this automated planning approach to other disease sites. Examples include lung treatments, which often require partial arcs, simultaneous integrated boost for the pelvis, necessitating rapid dose falloff between different target volumes, and head and neck with a large number of OARs to consider. Some of these sites are currently under investigation.

## 5 CONCLUSIONS

This study successfully demonstrated the development and implementation of an MCO‐based automated planning tool for clinical use in prostate and whole pelvis radiotherapy. The tool effectively standardized the planning processes from beam parameters such as arc, couch, and collimator angles to tradeoffs between the tumor coverage and dose to OAR, reduced dosimetrists’ workload, allowing generation of clinically acceptable plans that require only minor adjustments. A key finding of the study is that it highlights a safe, steady, and iterative process for deploying automated planning solutions, emphasizing the value of continuous improvement based on direct dosimetrists feedback throughout an extensive pilot phase. This study supports the integration of MCO‐based auto‐planning into routine clinical workflow to enhance efficiency and consistency of treatment plans.

## AUTHOR CONTRIBUTIONS

**Kai Huang**: Conceptualization; data curation; formal analysis; investigation; methodology; project administration; software; validation; visualization; writing—original draft; writing—review and editing. **Kai Wang**: Conceptualization; data curation; investigation; methodology; validation; writing—original draft; writing—review and editing. **Adam Schrum**: Validation; writing—review and editing. **Eric Kusmaul**: Validation; writing—review and editing. **Erica Fisler**: Validation; writing—review and editing. **Mariana Guerrero**: Conceptualization; data curation; formal analysis; investigation; methodology; project administration; validation; writing—original draft; writing—editing.

## CONFLICT OF INTEREST STATEMENT

The authors declare no conflicts of interest.

## ETHICS STATEMENT

This study was approved by our Institution's IRB (HP‐00108835) and it was conducted following all guidelines of ethics and patients’ privacy required.

## DECLARATION STATEMENT

During the preparation of this work the authors used generative AI—Gemini to check grammar and improve readability and language. After using this tool, the authors reviewed and edited the content as needed and took full responsibility for the content of the publication.

## Supporting information



Supporting Data

## Data Availability

Data is not available for sharing.
