# Supplementary material for: Development and clinical deployment of an automated planning tool for prostate only and male whole pelvis plans based on multi‐criteria optimization
Source: J Appl Clin Med Phys. 2026 May 4;27(5):e70598. doi: 10.1002/acm2.70598 (PMC13137941; doi:10.1002/acm2.70598)
Supplement: Supplementary file 1 — Supporting Data [file ACM2-27-e70598-s001.zip › 2025-08686-sup-0005-Supplemental_Material-S03.docx]

**Table S3**

| **ROI** | **Metrics** | **MCO mean** | **MCO stdev** | **Clinical mean** | **Clinical stdev** | **P value** | **Significance** | **n** |
| --- | --- | --- | --- | --- | --- | --- | --- | --- |
| **PTV** | **Average Dose (cGy)** | 4576 | 30 | 4576 | 30 | 0.844 | NS | 8 |
|  | **D90.0%[%]** | 100 | 1 | 99 | 1 | 0.055 | NS | 8 |
|  | **D90.0%[cGy]** | 4499 | 31 | 4476 | 45 | 0.055 | NS | 8 |
|  | **D95.0%[%]** | 99 | 1 | 98 | 1 | 0.078 | NS | 8 |
|  | **D95.0%[cGy]** | 4446 | 31 | 4414 | 59 | 0.078 | NS | 8 |
|  | **Max Dose (cGy)** | 4839 | 40 | 4786 | 36 | 0.023 | * | 8 |
|  | **Min Dose (cGy)** | 3472 | 117 | 3576 | 299 | 0.383 | NS | 8 |
| **CTV** | **Average Dose (cGy)** | 4599 | 30 | 4610 | 27 | 0.023 | * | 8 |
|  | **Max Dose (cGy)** | 4824 | 37 | 4781 | 37 | 0.039 | * | 8 |
|  | **Min Dose (cGy)** | 4368 | 37 | 4346 | 78 | 1.000 | NS | 8 |
| **Prostate** | **Average Dose (cGy)** | 4596 | 31 | 4611 | 31 | 0.078 | NS | 8 |
|  | **D95.0%[%]** | 101 | 1 | 101 | 1 | 0.023 | * | 8 |
|  | **D95.0%[cGy]** | 4546 | 31 | 4567 | 32 | 0.023 | * | 8 |
|  | **Max Dose (cGy)** | 4701 | 35 | 4715 | 37 | 0.383 | NS | 8 |
|  | **Min Dose (cGy)** | 4483 | 44 | 4516 | 35 | 0.023 | * | 8 |
| **Rectum** | **Average Dose (cGy)** | **2673** | 300 | 2940 | 196 | 0.008 | ** | 8 |
|  | **D0.03cc[%]** | 105 | 1 | 105 | 1 | 0.109 | NS | 8 |
|  | **D0.03cc[cGy]** | 4740 | 47 | 4714 | 31 | 0.109 | NS | 8 |
|  | **Max Dose (cGy)** | 4757 | 46 | 4732 | 30 | 0.148 | NS | 8 |
|  | **Min Dose (cGy)** | **330** | 194 | 447 | 318 | 0.008 | ** | 8 |
|  | **V3400cGy[%]** | 38 | 6 | 42 | 7 | 0.313 | NS | 8 |
|  | **V3700cGy[%]** | 34 | 6 | 36 | 7 | 0.641 | NS | 8 |
|  | **V4000cGy[%]** | 29 | 6 | 30 | 6 | 0.844 | NS | 8 |
|  | **V4300cGy[%]** | 22 | 6 | 21 | 4 | 0.547 | NS | 8 |
| **Bladder** | **Average Dose (cGy)** | **3021** | 440 | 3313 | 523 | 0.016 | * | 8 |
|  | **D0.03cc[%]** | 106 | 1 | 105 | 1 | 0.313 | NS | 8 |
|  | **D0.03cc[cGy]** | 4752 | 41 | 4732 | 40 | 0.313 | NS | 8 |
|  | **Max Dose (cGy)** | 4763 | 45 | 4740 | 42 | 0.250 | NS | 8 |
|  | **Min Dose (cGy)** | **942** | 267 | 1409 | 520 | 0.023 | * | 8 |
|  | **V3700cGy[%]** | 39 | 14 | 42 | 16 | 0.250 | NS | 8 |
|  | **V4000cGy[%]** | 35 | 13 | 36 | 14 | 0.383 | NS | 8 |
|  | **V4300cGy[%]** | 29 | 12 | 29 | 13 | 1.000 | NS | 8 |
|  | **V4500cGy[%]** | 23 | 10 | 22 | 12 | 0.547 | NS | 8 |
| **Femur_Head_L** | **Average Dose (cGy)** | **1004** | 75 | 1275 | 150 | 0.016 | * | 7 |
|  | **Max Dose (cGy)** | 3056 | 305 | 3069 | 289 | 1.000 | NS | 7 |
|  | **Min Dose (cGy)** | **68** | 16 | 91 | 26 | 0.047 | * | 7 |
| **Femur_Head_R** | **Average Dose (cGy)** | **974** | 141 | 1225 | 222 | 0.016 | * | 7 |
|  | **Max Dose (cGy)** | 2994 | 291 | 3047 | 340 | 0.375 | NS | 7 |
|  | **Min Dose (cGy)** | **67** | 13 | 95 | 24 | 0.031 | * | 7 |
| **Bowel_Small** | **V1500cGy[cc]** | 172 | 136 | 242 | 185 | 0.063 | NS | 5 |
|  | **V4000cGy[%]** | 16 | 22 | 16 | 18 | 0.625 | NS | 5 |
|  | **V4500cGy[%]** | 7 | 10 | 6 | 6 | 1.000 | NS | 5 |
|  | **V4500cGy[cc]** | 7 | 5 | 7 | 5 | 0.813 | NS | 5 |
|  | **Average Dose (cGy)** | **2065** | 765 | 2339 | 637 | 0.047 | * | 7 |
|  | **Max Dose (cGy)** | 4729 | 56 | 4704 | 37 | 0.297 | NS | 7 |
|  | **Min Dose (cGy)** | 485 | 303 | 559 | 399 | 0.219 | NS | 7 |
| **Bowel_Large** | **D0.03cc[%]** | 105 | 1 | **104** | 0 | 0.031 | * | 7 |
|  | **D0.03cc[cGy]** | 4712 | 27 | **4687** | 22 | 0.031 | * | 7 |
|  | **V3400cGy[%]** | 20 | 8 | 23 | 9 | 0.469 | NS | 7 |
|  | **V3700cGy[%]** | 17 | 7 | 18 | 9 | 0.469 | NS | 7 |
|  | **V4000cGy[%]** | 13 | 6 | 14 | 8 | 0.813 | NS | 7 |
|  | **V4300cGy[%]** | 9 | 5 | 10 | 6 | 0.688 | NS | 7 |
|  | **Average Dose (cGy)** | **1999** | 460 | 2341 | 339 | 0.008 | ** | 8 |
|  | **Max Dose (cGy)** | 4729 | 34 | 4703 | 18 | 0.250 | NS | 8 |
|  | **Min Dose (cGy)** | 338 | 127 | 419 | 241 | 0.078 | NS | 8 |

**Table S3:** The dose metrics (mean and standard deviations) for whole pelvis plans with fractionation of 4500 cGy in 25 fractions. The column n represents the number of plans with the corresponding ROIs. NS means not significant. * means P value is less than 0.05. ** means that P value is less than 0.01. *** mean that p value is less than 0.001. SV means seminal vesicles. The ones with better numbers are highlighted.
