# Supplementary material for: Development and clinical deployment of an automated planning tool for prostate only and male whole pelvis plans based on multi‐criteria optimization
Source: J Appl Clin Med Phys. 2026 May 4;27(5):e70598. doi: 10.1002/acm2.70598 (PMC13137941; doi:10.1002/acm2.70598)
Supplement: Supplementary file 1 — Supporting Data [file ACM2-27-e70598-s001.zip › 2025-08686-sup-0002-SI_Figure-S01.docx]

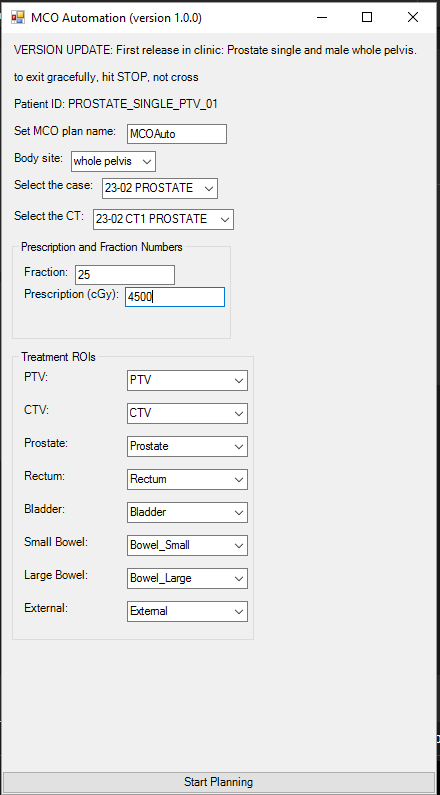


**Figure S1.** Graphical user interface. The users are able to specify plan name, body sites, CT, prescription and fraction numbers, and select ROIs.
