# Supplementary material for: Development and clinical deployment of an automated planning tool for prostate only and male whole pelvis plans based on multi‐criteria optimization
Source: J Appl Clin Med Phys. 2026 May 4;27(5):e70598. doi: 10.1002/acm2.70598 (PMC13137941; doi:10.1002/acm2.70598)
Supplement: Supplementary file 1 — Supporting Data [file ACM2-27-e70598-s001.zip › 2025-08686-sup-0003-Supplemental_Material-S01.docx]

**Table S1**

| **ROI** | **Metrics** | **MCO mean** | **MCO stdev** | **Clinical mean** | **Clinical stdev** | **P value** | **Significance** | **n** |
| --- | --- | --- | --- | --- | --- | --- | --- | --- |
| **PTV** | **Average Dose (cGy)** | 6060 | 23 | 6060 | 23 | 0.922 | NS | 10 |
|  | **D90.0%[cGy]** | 5980 | 33 | 5986 | 53 | 0.922 | NS | 10 |
|  | **D95.0%[cGy]** | 5905 | 41 | 5920 | 84 | 0.846 | NS | 10 |
|  | **Max Dose (cGy)** | 6346 | 49 | **6225** | 54 | 0.002 | ** | 10 |
|  | **Min Dose (cGy)** | 5013 | 130 | 5175 | 313 | 0.193 | NS | 10 |
| **Prostate** | **Average Dose (cGy)** | 6075 | 23 | 6083 | 19 | 0.322 | NS | 10 |
|  | **D95.0%[cGy]** | 6036 | 22 | **6053** | 18 | 0.006 | ** | 10 |
|  | **Max Dose (cGy)** | 6222 | 39 | **6168** | 26 | 0.010 | ** | 10 |
|  | **Min Dose (cGy)** | 5961 | 71 | 5982 | 61 | 0.375 | NS | 10 |
| **SV** | **Average Dose (cGy)** | 6083 | 21 | 6082 | 19 | 1.000 | NS | 10 |
|  | **D95.0%[cGy]** | 6038 | 21 | **6049** | 19 | 0.027 | * | 10 |
|  | **Max Dose (cGy)** | 6213 | 32 | **6162** | 28 | 0.002 | ** | 10 |
|  | **Min Dose (cGy)** | 5988 | 43 | **6013** | 25 | 0.049 | * | 10 |
| **Rectum** | **Average Dose (cGy)** | **2321** | 486 | 2483 | 523 | 0.020 | * | 10 |
|  | **Max Dose (cGy)** | 6159 | 107 | 6147 | 65 | 0.625 | NS | 10 |
|  | **Min Dose (cGy)** | **187** | 88 | 204 | 101 | 0.002 | ** | 10 |
|  | **V4080cGy[%]** | 13 | 5 | 13 | 5 | 0.557 | NS | 10 |
|  | **V4860cGy[%]** | 7 | 4 | 8 | 4 | 0.695 | NS | 10 |
|  | **V5280cGy[%]** | 5 | 4 | 5 | 4 | 0.492 | NS | 10 |
|  | **V5700cGy[%]** | **3** | 3 | 4 | 3 | 0.037 | * | 10 |
|  | **V6000cGy[%]** | 1 | 1 | 2 | 1 | 0.064 | NS | 10 |
| **Bladder** | **Average Dose (cGy)** | **2126** | 688 | 2437 | 799 | 0.010 | ** | 10 |
|  | **D0.03cc[cGy]** | 6292 | 32 | **6180** | 46 | 0.002 | ** | 10 |
|  | **Max Dose (cGy)** | 6309 | 28 | **6188** | 52 | 0.002 | ** | 10 |
|  | **Min Dose (cGy)** | **195** | 200 | 317 | 433 | 0.002 | ** | 10 |
|  | **V4080cGy[%]** | 18 | 7 | 20 | 7 | 0.275 | NS | 10 |
|  | **V4860cGy[%]** | 13 | 5 | 14 | 5 | 0.131 | NS | 10 |
|  | **V6000cGy[%]** | 5 | 2 | 6 | 2 | 0.375 | NS | 10 |
| **Femur_Head_L** | **Average Dose (cGy)** | **991** | 131 | 1568 | 215 | 0.002 | ** | 10 |
|  | **Max Dose (cGy)** | **2719** | 260 | 3190 | 154 | 0.004 | ** | 10 |
|  | **Min Dose (cGy)** | **47** | 24 | 84 | 53 | 0.002 | ** | 10 |
| **Femur_Head_R** | **Average Dose (cGy)** | **1009** | 114 | 1459 | 148 | 0.002 | ** | 10 |
|  | **Max Dose (cGy)** | **2678** | 318 | 2973 | 285 | 0.004 | ** | 10 |
|  | **Min Dose (cGy)** | **42** | 25 | 74 | 42 | 0.002 | ** | 10 |
| **Penile bulb** | **Average Dose (cGy)** | **1745** | 1505 | 2121 | 1300 | 0.039 | * | 8 |
|  | **Max Dose (cGy)** | **2929** | 2152 | 3996 | 1948 | 0.008 | ** | 8 |
|  | **Min Dose (cGy)** | 804 | 671 | 720 | 350 | 0.742 | NS | 8 |
|  | **V4000cGy[%]** | 16 | 25 | 19 | 24 | 0.312 | NS | 8 |

**Table S1** Dose metrics (mean and standard deviations) for prostate only plans with fractionation of 6000 cGy in 20 fractions. The column n represents the number of plans with the corresponding ROIs. NS means not significant. * means P value is less than 0.05. ** means that P value is less than 0.01. *** mean that p value is less than 0.001. SV means seminal vesicle. The ones with better numbers are in bold.
