# Supplementary material for: Development and clinical deployment of an automated planning tool for prostate only and male whole pelvis plans based on multi‐criteria optimization
Source: J Appl Clin Med Phys. 2026 May 4;27(5):e70598. doi: 10.1002/acm2.70598 (PMC13137941; doi:10.1002/acm2.70598)
Supplement: Supplementary file 1 — Supporting Data [file ACM2-27-e70598-s001.zip › 2025-08686-sup-0004-Supplemental_Material-S02.docx]

**Table S2**

| **ROI** | **Metrics** | **MCO mean** | **MCO stdev** | **Clinical mean** | **Clinical stdev** | **P value** | **Significance** | **n** |
| --- | --- | --- | --- | --- | --- | --- | --- | --- |
| **PTV** | **Average Dose (cGy)** | 7091 | 26 | 7091 | 26 | 0.469 | NS | 7 |
|  | **D90.0%[cGy]** | 6993 | 16 | 7021 | 31 | 0.016 | * | 7 |
|  | **D95.0%[cGy]** | 6911 | 20 | 6962 | 65 | 0.047 | * | 7 |
|  | **Max Dose (cGy)** | 7384 | 74 | 7257 | 92 | 0.016 | * | 7 |
|  | **Min Dose (cGy)** | 5866 | 176 | 6112 | 649 | 0.297 | NS | 7 |
| **Prostate** | **Average Dose (cGy)** | 7113 | 32 | 7110 | 34 | 0.219 | NS | 7 |
|  | **D95.0%[cGy]** | 7071 | 25 | 7072 | 25 | 0.938 | NS | 7 |
|  | **Max Dose (cGy)** | 7277 | 66 | 7209 | 87 | 0.031 | * | 7 |
|  | **Min Dose (cGy)** | 6965 | 78 | 6997 | 66 | 0.109 | NS | 7 |
| **SV** | **Average Dose (cGy)** | 7119 | 36 | 7115 | 32 | 0.313 | NS | 5 |
|  | **D95.0%[cGy]** | 7071 | 32 | 7074 | 21 | 0.625 | NS | 5 |
|  | **Max Dose (cGy)** | 7263 | 31 | 7216 | 68 | 0.313 | NS | 5 |
|  | **Min Dose (cGy)** | 7001 | 44 | 7030 | 28 | 0.125 | NS | 5 |
| **Rectum** | **Average Dose (cGy)** | 2482 | 459 | 2600 | 537 | 0.219 | NS | 7 |
|  | **D0.03cc[cGy]** | 7149 | 175 | 7134 | 94 | 0.578 | NS | 7 |
|  | **D15.0%[cGy]** | 4849 | 898 | 4892 | 943 | 0.688 | NS | 7 |
|  | **D20.0%[cGy]** | 4315 | 766 | 4268 | 674 | 1.000 | NS | 7 |
|  | **D25.0%[cGy]** | 3879 | 579 | 3781 | 509 | 0.813 | NS | 7 |
|  | **D35.0%[cGy]** | 3216 | 439 | 3238 | 446 | 1.000 | NS | 7 |
|  | **D50.0%[cGy]** | 2258 | 700 | 2472 | 664 | 0.297 | NS | 7 |
|  | **Max Dose (cGy)** | 7209 | 128 | 7166 | 83 | 0.156 | NS | 7 |
|  | **Min Dose (cGy)** | 134 | 84 | 144 | 104 | 0.469 | NS | 7 |
|  | **V4000cGy[%]** | 22 | 6 | 22 | 8 | 1.000 | NS | 7 |
|  | **V6500cGy[%]** | 5 | 5 | 6 | 5 | 0.578 | NS | 7 |
|  | **V7000cGy[cc]** | 4 | 6 | 4 | 6 | 0.688 | NS | 7 |
| **Bladder** | **Average Dose (cGy)** | 1442 | 667 | 1784 | 829 | 0.016 | * | 7 |
|  | **D0.03cc[cGy]** | 7199 | 151 | 7161 | 93 | 0.297 | NS | 7 |
|  | **D30.0%[cGy]** | 1737 | 1007 | 2235 | 1249 | 0.016 | * | 7 |
|  | **D35.0%[cGy]** | 1451 | 870 | 1957 | 1187 | 0.016 | * | 7 |
|  | **D50.0%[cGy]** | 744 | 573 | 1295 | 1089 | 0.031 | * | 7 |
|  | **D90.0%[cGy]** | 172 | 103 | 233 | 150 | 0.016 | * | 7 |
|  | **Max Dose (cGy)** | 7261 | 132 | 7190 | 89 | 0.016 | * | 7 |
|  | **Min Dose (cGy)** | 86 | 55 | 98 | 62 | 0.047 | * | 7 |
|  | **V4000cGy[%]** | 10 | 8 | 13 | 10 | 0.016 | * | 7 |
|  | **V6500cGy[%]** | 4 | 4 | 4 | 4 | 0.078 | NS | 7 |
|  | **V7000cGy[cc]** | 6 | 6 | 6 | 6 | 0.156 | NS | 7 |
| **Bowel_Large** | **D0.03cc[%]** | 43 | 34 | 55 | 41 | 0.063 | NS | 6 |
|  | **D0.03cc[cGy]** | 2981 | 2366 | 3847 | 2841 | 0.063 | NS | 6 |
|  | **D15.0%[cGy]** | 355 | 267 | 436 | 377 | 0.156 | NS | 6 |
|  | **D25.0%[cGy]** | 242 | 174 | 277 | 227 | 0.156 | NS | 6 |
|  | **D35.0%[cGy]** | 175 | 117 | 200 | 162 | 0.438 | NS | 6 |
|  | **D50.0%[cGy]** | 97 | 68 | 103 | 72 | 0.438 | NS | 6 |
| **Femur_Head_L** | **Average Dose (cGy)** | 1312 | 113 | 2051 | 304 | 0.016 | * | 7 |
|  | **Max Dose (cGy)** | 3331 | 226 | 3878 | 604 | 0.219 | NS | 7 |
|  | **Min Dose (cGy)** | 104 | 57 | 149 | 68 | 0.016 | * | 7 |
| **Femur_Head_R** | **Average Dose (cGy)** | 1233 | 137 | 2050 | 420 | 0.062 | NS | 6 |
|  | **Max Dose (cGy)** | 3063 | 197 | 3665 | 626 | 0.312 | NS | 6 |
|  | **Min Dose (cGy)** | 121 | 76 | 176 | 84 | 0.031 | * | 6 |
| **Penile bulb** | **Average Dose (cGy)** | 2886 | 2556 | 3193 | 2415 | 0.031 | * | 6 |

**Table S2.** The dose metrics (mean and standard deviations) for prostate only plans with fractionation of 7000 cGy in 28 fractions. The column n represents the number of plans with the corresponding ROIs. NS means not significant. * means P value is less than 0.05. ** means that P value is less than 0.01. *** mean that p value is less than 0.001. SV means seminal vesicle. The ones with better numbers are highlighted.
